# Optimization Design of Short Life Cycle Product Logistics Supply Chain Scheme Based on Support Vector Machine

**DOI:** 10.1155/2022/2311845

**Published:** 2022-09-28

**Authors:** Foshang Li

**Affiliations:** School of Business, Jilin Business and Technology College, Jilin, Changchun 130507, China

## Abstract

In order to better solve the problem of product logistics supply chains with short life cycles, a solution optimization of short life cycle product logistics supply chains based on support vector machines is proposed. This method recommends key technical problems and solutions through information represented by support vector machines and explore the research of short life cycle products to realize logistics supply chains. The research shows that, whether it is a retail channel or a network channel, the RMSE value of the effect index predicted by SVM is smaller than the RMSE value of the improved Bass. It can be seen that the SVM demand forecasting model constructed by considering multiple input factors can obtain a more accurate forecasting effect. The accuracy of the demand forecasting model based on SVM is verified.

## 1. Introduction

In recent years, the rapid development of the e-commerce industry not only drives the rapid development of the traditional consumption field but also gradually forms the dual-channel supply chain mode of coexistence of the traditional industry and the e-commerce platform. The rapid development of e-commerce directly promotes the rapid development of the traditional logistics industry. In addition, the continuous progress of science and technology, the transformation and rise of personalized demand of market consumer groups have virtually promoted the upgrading of products, and at the same time, the life cycle of products is constantly shortened; that is, short life cycle products have begun to appear. The market demand of this kind for product is too uncertain and time-sensitive, which virtually increases the difficulty of the supply chain management of enterprises. Enterprise supply chain management requires the mutual activities of different entities and related functions to gradually achieve a high degree of coordination, and on the basis of a high degree of coordination and cooperation, gradually achieve the purpose of a more flexible and low-cost corresponding consumer market [1, 2]. Therefore, the product supply chain with a short life cycle must be further optimized. On this basis, a model prediction algorithm based on support vector machine and random inventory control adjustment and an optimization strategy based on TOC dynamic buffer management advantage are proposed, and this model is applied to the short life cycle product logistics supply chain to optimize the supply chain scheme [3]. Due to the fashion, seasonality, and depreciation characteristics of short life cycle products, it is particularly important to accurately predict logistics supply chain information and quickly respond to market changes. Traditional time series and other forecasting methods need to find out the law of historical data changing with time to predict market demand, but this method lacks consideration of short product life cycles and lacks historical data. Some scholars take into account the characteristics of short life cycle products and use the Bass model family to achieve prediction, but there is a lack of research on the different characteristics of traditional and network channels. The support vector machine (SVM) method has significant advantages in solving the problem of small sample and nonlinear high-latitude model construction, and can improve the generalization ability of the model. Therefore, the use of SVM for forecasting has lower requirements on the amount of data, and is suitable for demand forecasting of products with short life cycles. Combining the characteristics of short life cycle product supply chain dual-channel sales cycle and less historical sales data, this paper discusses a short life cycle product demand forecast model based on Bass model that only considers the single factor of sales historical data; analyzes service and purchase intentions Bass improvement model under two factors. Based on this, the SVM algorithm is applied to the demand forecast of short life cycle products under dual-channel, considering the influence of price, season, promotion, industry status, historical sales data, and channel type of the short life cycle products in dual-channel sales. Combining the forecast correction value of the Bass model family with various influencing factors such as channels, a demand forecast model based on SVM is constructed, and simulation analysis shows that this method can improve the effect of short life cycle product dual-channel supply chain demand forecasting.

## 2. Related Works

Due to the rapid change in the market environment and various internal problems in the supply chain, the supply chain system must deal with various possible uncertainties in supply and demand in an appropriate way. At the same time, due to the inventory in the supply chain system, between the link between the medium and the buffer role. Therefore, good supply chain inventory management can effectively reduce the impact of various uncertainties on the overall supply chain system [4]. At the same time, in supply chain inventory management, a better management strategy and method should be able to ensure a higher level of customer service it can also maintain reasonable and effective control over the overall inventory level of the supply chain system, and increase the overall effective output of the system as much as possible, and further improve the comprehensive competitiveness of enterprises in the supply chain [5, 6]. Therefore, inventory management has always been one of the important issues in traditional supply chain research, and related research has been continuously developing. The existing inventory theory research can be roughly divided into deterministic inventory models, stochastic inventory models, multilevel inventory model, infinite cycle inventory models, and various inventory contract models which are currently in the research hotspot. For the deterministic inventory model, its main parameters, including the specific order quantity of each batch, order cost, and unit inventory cost, remain unchanged while the demand for related products is determined. In the research of this model framework, we generally conduct in-depth analysis and research from the perspectives of product quantity discount setting, replenishment lead time setting, and how to compensate for out-of-stock. In reality, there are many conditions, such as uncertain lead time, random demand, and variable ordering costs, and out-of-stock allowance. Therefore, the research on the stochastic inventory models has more practical significance [7].

Short life cycle products are commodities with a relatively short and fixed sales time, such as newspapers and magazines, fashion, all kinds of decorations, and increasingly electronic consumer goods [8]. For these short life products, distributors typically place orders well in advance of the selling season. It is also difficult for distributors to obtain particularly accurate demand information before placing orders. Therefore, only after calculating the expected revenue brought by all kinds of possible situations, such as shortage or overstocking, can it formulate the optimal ordering strategy by way of prediction according to the actual situation.

Considering that manufacturers' channel selection, channel competition, and contract coordination are the main factors affecting supply chain performance, some scholars put forward that perishable manufacturers' sales channel selection under the Internet environment will be affected by product price, the freshness of perishable products, and product price and quality of competitive channels. It is found that the increase of e-commerce channels is beneficial to the profit increase of the whole supply chain system. In the case of high distribution cost, the low wholesale price, and market demand fluctuation, it is more advantageous to choose two sales channels than a single channel. Considering the convenience factor of the channel, the pricing problem of the two sales channels is studied after the manufacturer adds the e-commerce channel to the two-layer supply chain system. The optimal coordinated price strategy of a dual-source supply chain is studied from the perspective of whether demand information is shared. The influence of service competition on channel pricing and profit distribution in the dual-channel supply chain is studied. From the perspective of consumers' sensitivity to service quality and price, different coordination modes of supply chain dual-source channels are studied. This article studies the win-win problem of supply chain channels achieved by manufacturers through quantity discount contracts in a mixed channel supply chain systems [9, 10].

## 3. Methods

### 3.1. Support Vector Machine Classification Algorithm

**Figure 1:**
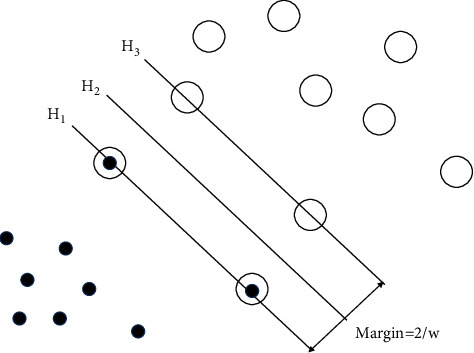
Optimal classification surface.

In the case of linear separability, a support vector machine is proposed based on the optimal classification hyperplane of a binary classification problem [11]. For example, if we look at two separate classes of problems on a two-dimensional plane, the basic idea can be expressed as a two-dimensional planar graph (see Figure 1). It is easy to observe that the two categories are divided into many possible boundaries, and each boundary has a related edge. If we choose the one with the maximum interval, there is little possibility of mistakes in the classification of unknown items in the future. Support vector machines map samples from input space to higher dimensional space by the kernel function. In this way, the samples on both sides of the dividing boundary can be accurately split, and the optimal classification plane can be found to maximize the classification distance between the two categories [12]. The empirical risk Remp(*w*) = 0 and the confidence range *φ* (*h*/*n*) is minimized, so that the real risk *R*(*w*) is minimized.

Assume that the given sample set is shown in:

(1) $$\begin{array}{c} \left\{\left(x_{1},y_{1}\right),\left(x_{2},y_{2}\right),\ldots ,\left(x_{n},y_{n}\right)\right\}\text{,}\\ x\in R^{d}\text{,}\\ y\in \left\{1,-1\right\}\text{.} \end{array}$$

The two types of samples in the figure above are represented by hollow points and solid points, respectively. *H*2 is the classification line, i.e. there is no boundary line separating the two types incorrectly, and the width of *H*2 and *H*3 lines is the classification interval [13].。The normalized processing, equation *w*·*x* + *b* = 0 for the equation of classification line, in high dimensional feature space, can be calculated by the equation *H*2, *H*3 classification interval is 2/||*w*||. According to the theory in the previous section, in order to obtain the maximum interval hyperplane of a linearly separable problem, constraints should be satisfied as shown in equation:

(2) $$\begin{array}{c} y_{i}\left(w\cdot x_{i}+b\right)\geq 1\text{.} \end{array}$$

To maximize the interval △ under the precondition of satisfying the above conditions, it is equivalent to the following optimization problem as shown in equations (3) and (4):

(3) $$\begin{array}{c} \min_{wh}\frac{1}{2}\left\|w\right\|s.t\text{,} \end{array}$$

(4) $$\begin{array}{c} y_{i}\left(w\cdot x_{i}+b\right)\geq 1\text{,}i=1,\ldots ,l\text{.} \end{array}$$

The above equation is a typical linear constrained convex quadratic programming problem, which determines the unique maximum interval classification hyperplane. Its Lagrange function is shown in the following equation:

(5) $$\begin{array}{c} L\left(w,b,\alpha \right)=\frac{1}{2}{\left\|w\right\|}^{2}-\overset{l}{\sum }\alpha_{i}\left(y_{i}\left(w\cdot x_{i}+b\right)-1\right)\text{.} \end{array}$$

The variable *α*_*i*_is the Lagrange multiplier. The saddle-point control optimization problem of the Lagrange function*L*(*w*, *b*, *α*), which differentiates with respect to *b* and *w*. *L*(*w*, *b*, *α*) solved the differential equations of *w* and *b* respectively and made the results zero. Two optimization conditions could be obtained, as shown in equations (6) and (7):

(6) $$\begin{array}{c} Condition1:\frac{\partial L\left(w,b,\alpha \right)}{\partial w}=0\text{,} \end{array}$$

(7) $$\begin{array}{c} Condition2:\frac{\partial L\left(w,b,\alpha \right)}{\partial b}=0\text{.} \end{array}$$

Equation (8) can be obtained from condition 1:

(8) $$\begin{array}{c} w=\overset{l}{\underset{i=1}{\sum }}\alpha_{i}y_{i}x_{i}\text{.} \end{array}$$

That is, the linear combination of training sample vectors is the weight vector of the optimal hyperplane. According to condition 2, the following equation can be obtained:

(9) $$\begin{array}{c} \overset{l}{\underset{i=1}{\sum }}\alpha_{i}y_{i}=0\text{.} \end{array}$$

Equation (8) problem can be transformed into a simpler “dual” problem by equation (9), as shown in the following equation:

(10) $$\begin{array}{c} W\left(\alpha \right)=\overset{l}{\underset{i=1}{\sum }}\alpha_{i}-\frac{1}{2}\overset{l}{\underset{i,j=1}{\sum }}\alpha_{i}\alpha_{j}y_{i}y_{j}\left(x_{i},x_{j}\right)\text{.} \end{array}$$

Equation (10) is the extremum problem of quadratic functions with inequality constraints. According to the optimality condition—Karush-Kühn-Tucke condition, the solution of the optimization problem of equation (10) must satisfy:

(11) $$\begin{array}{c} \alpha_{i}\left\{\left[\left(w\cdot x_{i}\right)+b\right]y_{i}-1\right\}=0\text{,}i=1,\ldots ,l\text{.} \end{array}$$

After solving the above quadratic programming problem, the classified decision function can be expressed asfollows:

(12) $$\begin{array}{c} f\left(x\right)=sgn\left(\overset{n}{\sum }\alpha_{i}*y_{i}\left(x_{i}\cdot x\right)+b^{*}\right)\text{.} \end{array}$$

When the training sample set is linear and not time-sharing, nonnegative relaxation variables can be introduced, as shown in:

(13) $$\begin{array}{c} \xi_{i}\text{,}i=1,2,\ldots ,l\text{.} \end{array}$$

The optimal problem of classified hyperplane is shown in:

(14) $$\begin{array}{c} \min_{w,b,\xi_{i}}\frac{1}{2}{\left\|w\right\|}^{2}+C\overset{1}{\underset{i=1}{\sum }}\xi_{i}\text{.} \end{array}$$

Solving the maximum value of the following function for *α* is its duality problem, as shown in equations (15)– and (17):

(15) $$\begin{array}{c} \overset{l}{\underset{i=1}{\sum }}\alpha_{i}-\frac{1}{2}\overset{l}{\underset{i,j=1}{\sum }}\alpha_{i}\alpha_{j}y_{i}y_{j}\left(x_{i},x_{j}\right)\text{,} \end{array}$$

(16) $$\begin{array}{c} y_{i}\left(w\cdot x_{i}+b\right)\geq 1-\xi_{i}\text{,}i=1,\ldots ,l\text{,} \end{array}$$

(17) $$\begin{array}{c} \overset{l}{\underset{i=1}{\sum }}y_{i}\alpha_{i}=0\text{,}\\ \begin{array}{c} i=1,\ldots ,l. \end{array} \end{array}$$

The performance of SVM is largely determined by the choice of penalty parameters and kernel parameters. In recent years, many scholars have focused on how to determine the parameters of kernel function to obtain the optimal generalization ability of SVM. At present, the simplest method is to first set the interval, range of each parameter, and search exhaustively within the defined step interval, and test one by one to get the parameter with the best performance on the learning object. In the application of the SVM model, the determination of the SVM model is determined by the determination of the kernel function. Therefore, the selection of kernel parameters is a very key step in the establishment of the SVM model. Although there is no universally accepted the best guideline for kernel selection, the Gaussian radial basis (RBF) kernel is the most commonly used kernel in many experiments and studies. Similarly, the radial basis kernel function (RBF) was selected as the kernel function of SVM. By analyzing the influence of radial basis parameters on the regression performance of the SVM model, the decision basis for the optimization of the SVM algorithm can be provided [14, 15].

### 3.2. Inventory Buffer Control Method in Supply Chain Coordination

#### 3.2.1. Questions Raised

**Figure 2:**
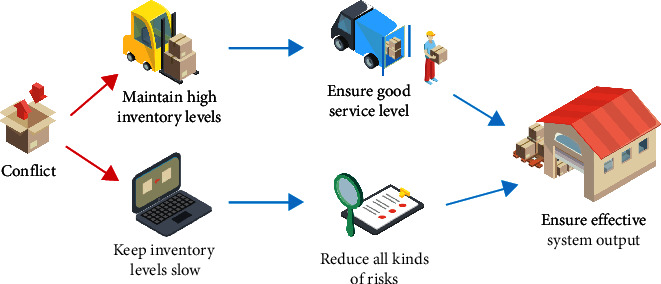
Inventory control system conflict diagram.

With the increasingly fierce market competition, customer preferences for short life cycle product supply chains are constantly changing. The increasing variety of commodities, the shorter life cycle of products, the frequent fluctuation of demand, and many other factors make the demand of each link of the whole supply chain show strong uncertainty and nonstability, and the uncertainty of demand greatly increases the difficulty of inventory control and management. According to the characteristics of short life cycle product supply chain, TOC puts forward methods and suggestions to establish a corresponding coordination system. The first is to take the VMI inventory system as the “link” between all the links in the supply chain, and apply the buffer management strategy in TOC, and then take this as the basis and main way of information transmission between each link. Therefore, in fact, in this supply chain system, the inventory system determines the overall “strength” of the supply chain system, so the setting of the inventory control system is particularly important for the short life cycle product supply chain based on the TOC system to be studied [16, 17]. The main contradictions commonly existing in various supply chain inventory control systems are shown in Figure 2.

#### 3.2.2. Problem Analysis

On the one hand, in order to ensure a good service level for the downstream links and reduce possible delivery delays or shortages, it is necessary to maintain a high inventory level of raw material parts or finished products in the supply chain system. On the other hand, upstream supply chain enterprises also need to reduce the backlog of raw material parts inventory or finished products, the occupation of capital and all kinds of risks brought by inventory. Therefore, it is necessary to keep a small inventory [18]. At the same time, it should be noted that in the case of nonstationary demand, the contradiction between inventory cost and production satisfaction rate is often more difficult to balance because the fluctuation of demand is difficult to be described by probability model. In order to ensure the effective output of the whole supply chain system, it is necessary to ensure the better service level of each link of the system and reduce the various risks faced by the supply chain system. The assumption of the upper part of the conflict diagram is that if the overall inventory of the supply chain keeps a high inventory level, good service level of the supply chain system can be guaranteed [19]. However, the current reality is that the coexistence of high inventory level and a low service level often leads to the shortage of urgently needed products in the market and the high inventory level of products with poor sales. Therefore, a higher inventory level cannot guarantee a good service level in the supply chain system. The assumptions therein are not tenable similarly, for the lower half of the conflict graph; it is assumed that if the overall inventory of the supply chain keeps a low inventory level, various risks caused by inventory can be reduced in the supply chain system [20]. Especially in the supply chain of fashion products, the demand volatility is often very large, especially in some seasonal peak demand; too low inventory level will make the supply chain system unable to meet the market demand, and then, have to face the risk of more severe market competition. Therefore, a low inventory level cannot completely reduce various risks in the supply chain system, and its hypothesis is also not valid. Based on the above analysis, in fact, the core problem of inventory control is still caused by the uncertainty of demand. If the inventory level is maintained at the appropriate time, the core conflict will be resolved, so the supply chain coordination needs a set of inventory control systems that can dynamically adjust the inventory level.

#### 3.2.3. Problem Solving

In order to carry out effective inventory control in supply chain coordination and solve the contradiction between inventory cost and customer service level, the following measures can be taken: first, improve the accuracy of demand prediction, make full use of historical demand data and expert knowledge provided by decision makers to make an effective prediction. Second, the inventory control strategy should have strong flexibility. When demand changes suddenly or seasonal demand comes, inventory control parameters can be flexibly adjusted according to historical information.

### 3.3. Construction of an Improved Bass Demand Forecast Model Based on Sales Records

The demand forecasting method, usually based on historical sales records, is to scientifically fit the past sales through a large number of historical sales records of products to find their demand trends. Due to the short sales period of short life cycle products, it is difficult to obtain a large number of historical sales records for a long period of time, usually only one year or even a few months of sales records. In response to this situation, this paper regards market sales as a process of continuous diffusion and penetration. That is, first, dealers attract a small number of consumers with a certain sense of innovation through product promotion or trial, and these consumers drive subsequent consumers, and so on. While a realistic diffusion process involves a large number of factors and their relationships, this complexity can be modeled and studied by the improved Bass diffusion model. Considering that the improved Bass model family has better applicability, the improved Bass model is firstly used to discuss the demand forecasting problem of short life cycle products, considering only the historical sales record as a single factor. The Improved Bass model is a model used to predict the sales of consumer goods. The improved Bass model can analyze the decision time of consumers to purchase products. It believes that the buyers of a product are influenced by external or internal factors, and thus, divides potential buyers of products into two categories: (1) innovation groups, which are susceptible to external influences, that is, mass media; (2)imitation groups, which class groups are susceptible to internal influence, that is, word-of-mouth influence. The core idea of the model is that the purchasing decision of the innovation group is independent of the purchasing behavior of other members of the social system, and the time when the imitation group purchases products is affected by the social system, and this influence increases with the increase of the number of buyers, thus, imitating the purchasing decision of the group. Time is influenced by members of the social system. According to the Bass thought above, let *f*(*t*) be the time density function of adopters in period *t*, indicating the possibility of purchasing in period *t*; *f*(*t*) is the proportion of cumulative adopters to all buyers in period *t*; *ρ* is the innovation group coefficient, indicating the external influence of the attributes of the communication product that can be easily verified, such as price, function, and media *ρ* ∈ (0, 1); The propaganda function of potential users, such as products, disseminates some characteristics of products that require long-term experience to discover, *q* ∈ (0, 1); *m* is the potential purchase amount; *n*(*t*) is the cumulative purchase amount in the *t* period; *n*(*t*) is the noncumulative purchase amount in period *t*; *X*(*i*) is the product demand.

### 3.4. Optimization Model of Short Life Cycle Product Logistics Supply Chain Scheme Based on Support Vector Machine

The demand forecasting method based on historical sales records only focuses on the sales records of products, so it has certain limitations. The demand forecasting method based on service-purchase intention takes into account the market behavior characteristics of participants in addition to the sales record data, and has made certain progress. However, these two schemes can only distinguish the two channels by their different historical sales record values, but ignore the unique characteristics of the two channels. To this end, this paper considers the dual-channel demand forecast for short life cycle products under multiple influencing factors. Multiinfluencing factors refer to the dual-channel market demand that is affected by multiple factors. For retail channels, it is mainly affected by product price, seasonal index, promotion level index, industry status quo, the number of people entering the store, and historical sales data; for online channels, it mainly considers product price, seasonal index, and promotion level index, industry status, credit index and historical sales data and other influencing factors. Considering that SVM can effectively analyze multiple small samples and multiple input factors, the SVM method is combined with the improved Bass diffusion model, and the product price, seasonal index, promotion degree index, industry status quo, number of shoppers, credit index, and historical sales are introduced. Data and other market-influencing factors are used, and the sales value predicted by the improved Bass model is used as the input vector to establish a dual-channel short life cycle product demand forecasting model under multiple influencing factors.

#### 3.4.1. Establish a Demand Forecasting Model Based on Support Vector Machine

According to the basic idea of SVM regression, through a nonlinear mapping Φ, the sample data (*x*_*i*_, *y*_*i*_) is mapped to the high-latitude feature space *F*, and linear regression is performed in this space:

(18) $$\begin{array}{c} f\left(x\right)=\left(\alpha^{T}\Phi \left(x\right)\right)+b,\Phi :R^{n}⟶F,\alpha \in F\text{.} \end{array}$$

The support vector machine regression can be expressed as the following constrained optimization problem, that is, the quadratic programming form of the objective function is

(19) $$\begin{array}{c} s,t.R^{T}\left[\begin{array}{c} \alpha \\ \alpha^{*} \end{array}\right]=0,0\leq \alpha_{i},\alpha_{i}\leq C. \end{array}$$

In the formula: *i* = 1, 2,…, *m*; *α* is the weight vector, *α*∈*F*; *Q* and *P* are the two specified vector groups. When *i* = 1, 2,…, *l*, *r*_*i*_ = 1; then, *i*=*l*+1, *l*+2,…, 2*l*, *r*_*i*_=−1. Among them, *x*_*i*_(*i*=1,2, ⋯, *l*) is the input of the ith training sample, *y*_*i*_(*i*=1,2,…, *l*) is the output of the ith training sample, write a program according to the above process, that is, get the regression decision function of the SVM demand forecasting model:

(20) $$\begin{array}{c} f\left(x\right)=\overset{l}{\underset{i=1}{\sum }}\left({\bar{\alpha }}_{i},{\bar{\alpha }}_{i}^{*}\right)K\left(x,x_{i}\right)+\bar{b}\text{.} \end{array}$$

#### 3.4.2. Determining SVM Optimization Parameters

The most commonly used method for SVM parameter optimization is to make the minimum penalty parameter *c* and the reciprocal *g* of the number of attributes in the input data that can achieve the highest verification classification accuracy take values within a certain range. For a certain set of *c* and *g*, the K-CV method is used to obtain the training set verification classification accuracy under the set of *c* and *g*, and finally, the set of *c* and *g* with the highest training set verification classification accuracy is selected as the best. Parameters. After the regression decision function is obtained, the normalized forecast sample is substituted into the regression decision equation, that is, the value *y* of the demand forecast between (0, 1) can be obtained, which can be converted into the actual forecast value by using the formula (20):

(21) $$\begin{array}{c} y=\hat{y}\left(y_{\max}-y_{\min}\right)+y_{\min}. \end{array}$$

In the above forecasting process, the fitted values, historical record values, and other market characteristics of the improved Bass model are substituted into the model in the form of input vectors, and the optimal parameters and demand forecast values can be determined through model simulation.

### 3.5. Dual-Channel Supply Chain Coupling Model for Short Life Cycle Products

#### 3.5.1. Supply Chain Model of Different Economic Forms

*(1) Supply Chain Model of Different Economic Forms Based on Customer Order Separation Point (CODP)*. For manufacturers, generally speaking, their internal supply chain operation is separated from customer orders, and there is a combination of design process, manufacturing process, assembly process, delivery process, and after-sales service process in the customer order completion process. Among them, the activity on the left of the customer order separation point is push type, and the activity on the right of the customer order separation point is a pull type. Therefore, it divides the supply chain into two parts: one is to respond directly to the needs of the end customer, and the other is to cushion the change of supply chain demand with planning and strategic inventory. The decoupling point is also the turning point of enterprise production activities from forecasted inventory production to customized production in response to customer demand. The equation and optimization of plans at this point is no longer based on prediction but on customer orders and resource allocation of the enterprise itself.

- *Engineer to Order (ETO)*. The needs of each consumer are different. Production can only be organized by designing products according to the needs of consumers when they receive orders, which requires a long time to prepare production technology. In this type of production, products are largely designed to meet the requirements of a particular consumer. To be designed to support consumerization is an important function and component of the production process. In this type of production, the production batches of products are small, but the design work and the final product are often very complex. In the production process, each job has to be specially handled so that the various subparts of a large product or project can be precisely matched at the final stage so that the different subparts produced by different people and different places can be combined into a complex product or project. Very advanced configuration systems are required for overall coordination and management control.
- *Make to Order (MTO)*. It purchases raw materials in advance according to product demand characteristics and forecast, and starts production after consumer demand is determined. The enterprise starts the manufacturing process after the order arrives, and the customer provides the design data, and the enterprise carries out personalized production according to the design requirements. Because customer orders trigger production, the finished product is rarely kept.
- *Assemble to Order (ATO)*. A product is a sequence of optional parts or prefinished components that users can select according to their individual needs. Manufacturers produce semifinished products or components, assemble them according to customer orders, and reduce lead times by producing standardized modules and assembling customized products to order. It also reduces the risk of early production. For example, computer production and assembly manufacturers cannot assemble a computer without receiving an order. However, in order to improve the response speed to consumers, they can produce some standard components in advance according to the market forecast and conduct rapid assembly when the orders arrive to meet the personalized needs of consumers.
- *Make to Stock (MTS)*. Consumers basically have no suggestions or requirements for the determination of final product specifications, and they have little input. Manufacturers do not make products tailored to any particular consumer. The products of the enterprise are manufactured before the order arrives, and the customer needs are satisfied through the inventory. The MTS production plan is based on a market forecast; such products should have relatively accurate forecast accuracy.
- *Physical Distribution on Stock (PDS)*. The demand for production comes from distributors. The product is standardized, and the manufacturer does not know who the final consumer is during the production process but predicts demand information based on historical sales data. This is generally a mass-consumer product, and management focuses on forecasting and inventory control.

**Figure 3:**
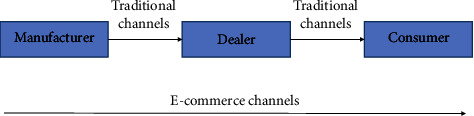
E-commerce dual-channel supply chain system.

*(2) Lean-Agile Supply Chain Coupling Based on Customer Order Separation Point (CODP)*. CODP is a certain point in the manufacturing process as well as a certain point in the supply chain, which is closely related to the enterprise supply chain. If the customer order separation point (CODP) of each link in a manufacturing enterprise is extended to a multistage supply chain, it involves suppliers, manufacturers, distributors, consumers, and other links. And for a supply chain system, its response time refers to the sum of time accumulated in all stages of the whole process, from the supply chain detecting the demand of the end customer and receiving the order to the delivery of the product/service to the end customer. Obviously, the response process of the supply chain to the final customer is actually the completion process, of the customer order, and its length depends on the point at which the customer order customization begins. In general, the part of direct response to consumer demand has strong demand correlation and autocorrelation, and it is easy to complete its relatively accurate prediction with a scientific algorithm through traditional forecasting methods and adopt the supply chain operation mode of MTS. The other part needs to use demand-side supply chain predictive management and adopt the supply chain operation mode of MTO. The dual-channel supply chain system of e-commerce is shown in Figure 3.

#### 3.5.2. The Delay Strategy is Coupled to the MTO/MTS Supply Chain

**Figure 4:**
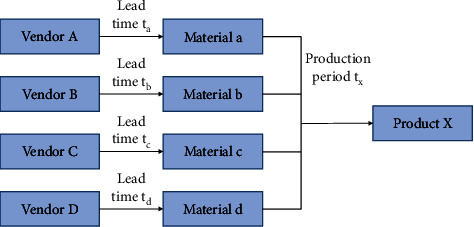
MRP material procurement model.

1. MTO/MTS supply chain coupling model from the perspective of flexible low-cost response. Lean supply chain management originates from lean production management and its bigger foothold lies in the reduction of supply chain costs. It refers to a series of supply chain planning, implementation, and control processes that optimize and transform the whole chain, including the upstream and downstream chains, to eliminate unnecessary steps, delays, waiting, and consumption, eliminate waste in enterprises, minimize costs, and meet consumer needs to the greatest extent. Lean supply chain management requires upstream and downstream efforts to reduce costs and waste throughout the process. See Table 1.
2. The competition of consumer demand response has developed from the simple quick delivery to the stage where enterprises unconditionally accept the increase, decrease, change or even cancel the demand put forward by consumers at any time. Enterprise cost management has also developed from the reduction of procurement, production, storage, transportation, and other physical costs to the total cost competition stage, including physical costs and market response costs, including out-of-sale costs and inventory holding costs. The above new supply chain competition stage generates a new supply chain development model—a flexible low-cost response model.
3. Combined with the above analysis and flexible low-cost response model, there is a coupling mechanism between lean supply chain and agile supply chain in enterprise operation. The advantages, disadvantages and complementarities of MTS-oriented supply chain strategy design in lean supply chain and MTO-oriented supply chain strategy design in agile supply chain are considered, and the factors and requirements of MTS supply chain in cost reduction and MTO supply chain in flexible response are combined.
4. MTO/MTS supply chain coupling model based on two-stage supply system. Generally speaking, manufacturing enterprises issue material purchase orders based on MRP operations and require all materials to be delivered at the same time specified. Material quantity conforms to BOM structure ratio of production product. In order to facilitate the study, this section selects a simple material procurement model consisting of only four raw materials and only one supplier for each raw material, is shown in Figure 4.

**Figure 5:**
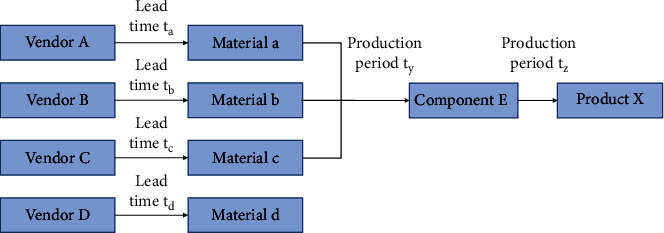
Delayed manufacturing model.

In operation, the materials required for production are often not all in accordance with BOM requirements. When the delivery time of products is tight, the manufacturer will start production activities based on all the materials in stock when the delivery time of the missing materials is relatively clear. This is not only the practice of advance production but also the concrete implementation of delayed production, as shown in Figure 5.

Through the second-order supply system, manufacturers can divide a wide variety of raw materials with different lead periods into multiple components or materials (raw materials) with strong generality, relatively easy demand prediction, and a long production cycle according to the process characteristics, and it adopts an MTS type supply chain to compress the order response speed of this part of component modules or materials, and it purchases and manufactures costs to start final product assembly after receiving consumer orders, which not only meets the rapid demand of consumers but also reduces the cost of the supply chain

The MTO/MTS supply chain coupling model of the above two-stage supply system is based on one premise. That is, before all kinds of materials arrive, part of the material has the technological basis of advanced production. Similarly, the quantity and type of each order are not consumed in the first place, which provides a management basis for the manufacturer's batch delivery

#### 3.5.3. MTO/MTS Supply Chain Coupling Model

**Figure 6:**
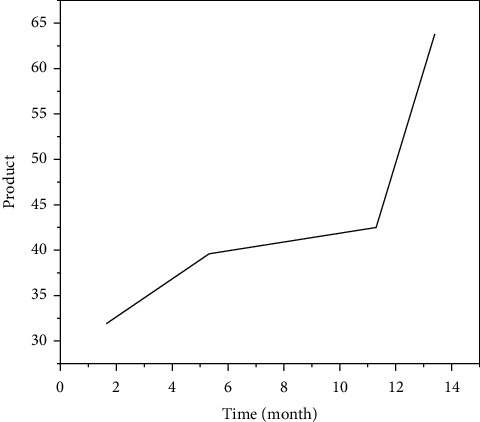
Classification of products (broken lines).

1. *Supply Chain Coupling Model Based on Customer Order Separation Point*. Time-dimension Customer Order Decoupling Point (TCODP) is used to describe the Time course from the Customer's Order to the customized product delivered to the end customer. It divides the whole supply chain into two parts: the order pull chain contributes (S2 chain), and the inventory push chain contributes (S1). This section takes the MTO/MTS supply chain with a short life cycle order as the research object, and adopts the link analysis method to decompose the supply chain into a process consisting of a series of five interactive links, including order processing, product design, procurement/supply, production and processing, and product distribution. See Table 2.
2. *MTO/MTS Supply Chain Coupling Model Considering Economic Value*. Decoupling Point (DP) refers to the demarcation Point in the MTO/MTS supply chain. The choice of Decoupling Point plays a key role in the rapid response of consumer demand and the reduction of supply chain costs. According to the analysis in the MTO/MTS supply chain coupling model from the perspective of flexible and low-cost response, the left side of the decoupling point is the push production mode based on prediction, and the right side is the pull production mode based on order. This point is likely to become the point of delayed manufacturing and order separation, and also the supply chain inventory control point. The economic comparison between forecast-driven and order-driven production is shown in Table 3:
3. Economic Value Analysis Dimension Analysis. This section will analyze the influencing factors of Decoupling Point from three dimensions: material versatility, customer demand diversity, and delivery time. Material versatility: refers to the extent to which materials can be used for different purposes and by different users, depending on the practicability of the product and the position of the Decoupling Point. When the material can meet the needs of multiple types of customers or when there is no market segmentation, its versatility is high; with the further processing of materials from raw materials to components (semifinished products) and then, to finished products, the universality of materials is gradually reduced.
4. *MTO/MTS Supply Chain Coupling Model Based on Short Life Cycle Products*. By jumping out of the limitations of traditional functional products and innovative products and just comparing different products, the classification of the same product under different demand-deterministic environments is expanded and expanded; the nature of the product is similar to functional products when its demand is determined and innovative products when its demand is uncertain. See Figure 6.

**Figure 7:**
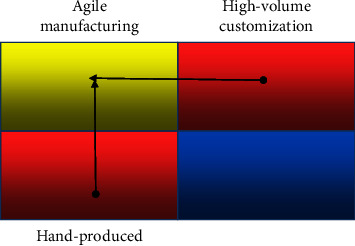
MTO/MTS supply chain coupling for product matching.

Mass procurement production and agile response are both designed to meet the different needs of consumers. They both produce customized products in the mode of flexible and low-cost response but adopt different technical routes. The maximum profit model of the supply chain is used to explain the response. They are the best choice to meet consumer demand under certain constraints. See Figure 7.

## 4. Results Analysis

Suppose a manufacturer sells a class of fashionable electronic products with a high replacement rate through dual channels. Among them, one channel is the traditional retail channel, and the other channel is to directly conduct transactions with end customers through its own e-commerce outlets. The sales volume and influencing factor indicators of retail channels and online channels are shown in Tables 4 and 5.

According to the basic Bass model constructed in Section 2 and the programming of the improved Bass model, combined with the optimization method, Matlab2010 is used to perform nonlinear fitting to improve the goodness of fit. First, the 14-month sales data of the retail channel are fitted, and the parameter values of the fitting curve equation can be obtained, as shown in Table 6.

As can be seen from Table 6, compared with the basic model of retail channels, the potential purchase volume of retail channels under the improved Bass model decreases slightly, and the coefficient of innovation group and imitation group increase slightly, which more accurately reflects the market demand of retail channels.

**Figure 8:**
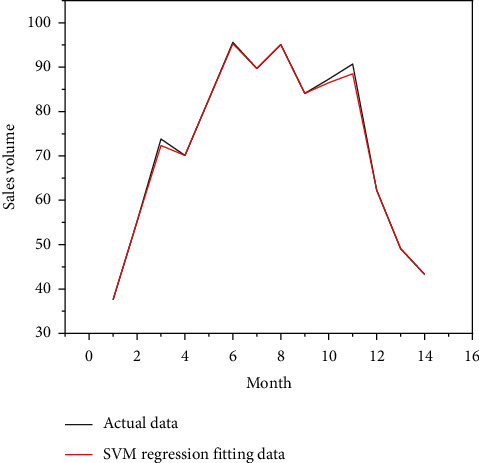
Data of SVM model retail channel sales.

**Figure 9:**
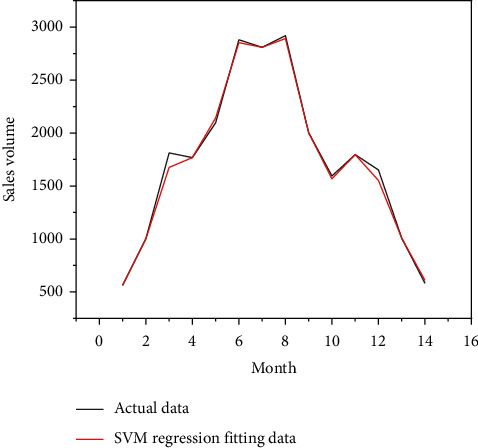
Data diagram of SVM model network channel sales fitting.

**Figure 10:**
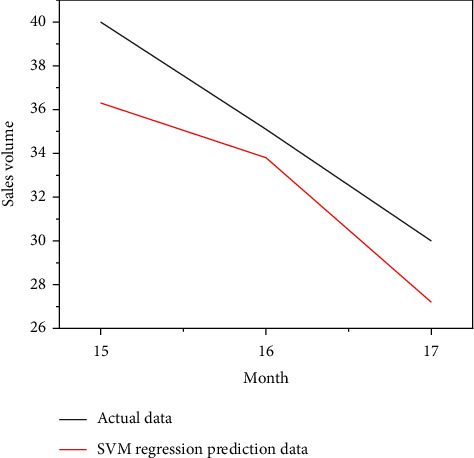
Sales forecast data diagram of SVM model retail channel.

**Figure 11:**
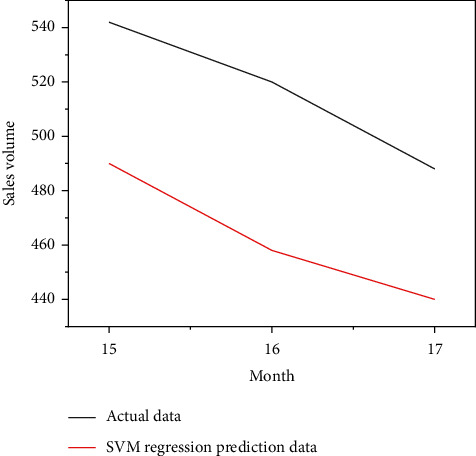
Sales forecast data of SVM model.

Put the data of the training group of retail channels and network channels into the SVM model for calculation, and put the data of the test group into the SVM model for prediction, and you get the comparison chart of the simulated sales volume and the actual sales volume of the SVM model for the first 14-months of retail channels and network channels, as shown in Figures 8 and 9, as well as the forecast sales and actual sales in the next three months, as shown in Figures 10 and 11.

Table 7 further compress the prediction effect index RMSE, that is, the regression root mean square, or the fitting standard deviation of the regression system. It can be seen from the table that, whether it is a retail channel or a network channel, the RMSE value of the effect index predicted by SVM is smaller than the RMSE value of the improved Bass. It can be seen that the SVM demand forecasting model constructed by comprehensively considering multiple input factors can obtain a more accurate forecasting effect.

According to the definition of dual channels mentioned above, whether it is traditional retail channels or online channels, the short life cycle products sold originate from manufacturers and ultimately flow to end customers. Obviously, the total market demand forecast is composed of the superposition of demand forecasts of two channels, that is, the forecasted sales volume from the dual-channel system.

## 5. Conclusions

Based on the work practice and literature research and according to the double channel supply chain ordering mode and the characteristics of short life cycle products, a comprehensive ordering optimization method and system are proposed. This method system can not only effectively solve the existing problems of short life cycle products in dual-channel supply chain ordering, but also enrich the content of dual-channel supply chain theory. The demand forecasting model is optimized and the demand forecasting model under the dual-channel supply chain is proposed. Traditional demand forecasting methods, such as quantitative forecasting method, the time series method, and causal analysis, assume that the law of demand in the past will remain unchanged in the future. With the rapid iteration of products with a short life cycle and the rapid change of consumer demand in the e-commerce environment, the accuracy of demand prediction is difficult to guarantee by traditional forecasting methods. In the existing literature, some scholars reduce the impact of demand uncertainty and improve the accuracy of demand prediction from the perspectives of repeated purchase, demand diffusion, increased order times, and demand renewal, respectively. However, the above studies did not consider the demand impact factors of products with short life cycle and the demand overlap and conflict brought by e-commerce dual channels. To this end, the article puts forward the characteristics of considering the influencing factors of product demand, the increase of demand information channels of dual-channel supply chain, the superposition effect of O_2_O demand interference and conflict, the change of O2O demand alone or coupled change, and deduces the demand prediction model of optimization of product demand factors and the O2O demand prediction model of dual-channel supply chain. At the same time, products are classified from the perspective of demand certainty, which provides a theoretical basis for the study of the MTO/MTS coupling orders.

## Figures and Tables

**Table 1:** Operation characteristics of lean supply chain and agile supply chain.

	Lean supply chain	Agile supply chain
Product design strategy	Achieve maximum performance with the lowest product cost	Provide solutions for customers, use a large number of common parts, and delay customer differentiation
The pricing strategy	The margin is low and price is the main factor to attract customers	Price is not the main attraction for customers
Production strategy	Reduce costs by increasing the utilization of equipment and personnel	Maintain production flexibility and try to meet uncertain demand
Inventory strategy	Realize the minimum inventory to array low-cost	Maintain safety inventory to meet uncertain demand
Lead-time strategy	On the basis of not increasing the cost, reduce the supply cycle	Maintain safety stock. To satisfy the uncertainty of demand
Supply strategy	Balance costs and service levels	Water in speed, elasticity, and service. Balance up
Transport strategy	More reliance on low-cost means of transportation	Rely more on block agility

**Table 3:** Comparison of forecast—driven and order—pulled production economics.

Project	Predict to promote	Order pull
*Starting point*	Reduce purchasing and production cost as much as possible with accurate forecast	Be flexible and responsive to customer needs and changes
*Location*	Vendor-coupling point	Coupling point—consumer
*Material characteristics*	Long procurement cycle, variety, and centralized procurement to reduce unit price and production into components	Species is less
*Processing requirements*	Specialized equipment and skilled workers are generally required	Low requirements for equipment and workers
*Supplier selection*	Cost and quality	Speed, elasticity, and mass
*Implementation method*	Standardization, process, and modular	Customized
*Economic benefits*	Low cost operation	The pursuit of fast response can lead to higher costs
*Advantages*	Material versatility	Targeted, to meet the needs of customization
*Disadvantages*	Response period length	Need an inventory to be a buffer

**Table 2:** Supply chain based on time dimension Decoupling Points.

Supply chain structure	Flow of the supply chain	Process involved in each link
*Manufacturers*	Product design	(1) Modular design of components (2) BOM design
	Purchasing supply	(1) Material procurement process in different cycles (2) Different material procurement and inventory strategies
	Order processing	(1) Order acceptance and review (2) Production planning and control
	Production and processing	(1) Production process route
	Shipment delivery	(1) Order priority
*Dealers*	Order processing	(1) Order acceptance and review
	Shipment delivery	(1) Order priority
*Consumers*	Demand forecasting	(1) Prediction based on historical requirements (2) Requirement change
	Purchase order	(1) Regular or quantitative ordering plan
	Warehouse inventory	(1) Daily inventory strategy and level (2) Product acceptance and storage management

**Table 4:** Retail channel sales indicators.

Month	Price	Seasonal factors	Promotional efforts	Industry form	The number of into the store	Sales volume
1	1299	0.562	1	0.98	4806	38
2	1499	0.835	0.8	0.94	4345	54
3	1499	1.123	0.8	0.95	4021	73
4	1499	1.062	0.8	0.9	3967	71
5	1499	1.259	0.8	0.95	3452	82
6	999	1.456	0.8	0.93	4089	95
7	999	1.366	0.8	0.88	4496	89
8	1499	1.442	0.3	0.84	4372	95
9	1499	1.275	0.3	0.74	3751	85
10	1499	1.318	0.3	0.78	4154	88
11	1299	1.382	0.2	0.76	4248	91
12	1299	0.941	0.1	0.74	3908	61
13	1299	0.742	0.1	0.74	3628	48
14	1299	0.623	0.1	0.67	3288	42

**Table 5:** Various indicators of online channel sales.

Month	Price	Seasonal factors	Promotional efforts	Industry form	The number of into the store	Sales volume
1	1299	0.389	1	0.99	0.98	577
2	1499	0.695	0.9	0.95	0.97	1023
3	1499	1.225	0.9	0.97	0.94	1815
4	1499	1.198	0.9	0.91	0.89	1772
5	1499	1.412	0.9	0.95	0.89	2088
6	999	1.938	1	0.94	0.84	2865
7	999	1.885	1	0.88	0.88	2783
8	1499	1.956	0.5	0.86	0.91	2897
9	1499	1.339	0.5	0.76	0.82	1978
10	1499	1.075	0.5	0.78	0.78	1588
11	1299	1.208	0.3	0.78	0.81	1759
12	1299	1.108	0.3	0.76	0.75	1639
13	1299	0.677	0.3	0.76	0.69	999
14	1299	0.242	0.3	0.69	0.72	575

**Table 6:** Parameter values of retail channel fitting sales curve equation.

	*m*	*p*	*q*	*β*	*γ*
Retail channel foundation Bass model	1148.88	0.05	0.24	—	—
Retail channel improvement in the Bass model	798.52	0.06	0.28	0.03	1.18

**Table 7:** Comparison of data forecasting effect indicators between retail channels and online channels.

Evaluating indicator	Improved the bass retail channels	The SVM retail channel	Evaluating indicator	Improved the bass network channels	The SVM network channel
RMSE	4.7442	1.5316	RMSE	92.2839	32.0485

## Data Availability

The data are available upon request to the corresponding author.
